# Implementation of Brachytherapy for Patients With Cervical Cancer in Ethiopia: A 3-Year Practice Report

**DOI:** 10.1200/GO.22.00407

**Published:** 2023-08-18

**Authors:** Yitbarek M. Kibret, Wondemagegnehu Tigeneh, Ahmedin Jemal, Eva J. Kantelhardt

**Affiliations:** ^1^ Oncology Department, Yekatit 12 Hospital Medical College, Addis Ababa, Ethiopia; ^2^ Global Health Working Group, Martin-Luther-University Halle-Wittenberg, Germany; ^3^ Oncology Department, Addis Ababa University, Addis Ababa, Ethiopia; ^4^ Surveillance and Health Service Research, American Cancer Society, Atlanta, GA; ^5^ Department of Gynaecology, Martin-Luther-University Halle-Wittenberg, Halle, Germany

## Abstract

**PURPOSE:**

Although cervical cancer is the second most commonly diagnosed cancer in Ethiopia, brachytherapy (BT) was not a component in patient treatment until 2015. The purpose of this study was to identify the patterns of utilization as well as to describe the practice of BT in Ethiopia.

**MATERIALS AND METHODS:**

A retrospective descriptive data analysis of 138 patients with cervical cancer treated with a curative potential using BT from 2015 to 2018 at Tikur Anbassa Specialized Hospital, which housed the only BT facility in Ethiopia during the study period.

**RESULTS:**

During the first 3-year period of BT service commencement, each year n = 37, n = 36, and n = 65 patients with cervical cancer were treated, respectively, with curative intention treatment. The median age of these 138 patients was 50 years (range, 22-75). All the patients were in International Federation of Gynecology and Obstetrics stage Ib–IIIb group, and stage IIb (66.4%) was the predominant. Majority (79%) of the patients were treated primarily with radiotherapy (RT), while 21% received RT after surgery. More than half of these patients (62%) received a total RT dose of 82 Gy in equivalent dose in 2 Gy fractions (EQD2), while the rest received a dose ranging from 76 to 86 Gy. Concurrent cisplatin with RT was given only for 36% of the patients for undocumented reasons. The overall treatment time including both external-beam RT and BT was greater than 8 weeks in 21% of the patients.

**CONCLUSION:**

The utilization of BT service increased gradually and BT enabled the delivery of a higher RT dose to patients with cervical cancer (mostly stage IIB). However, there was protracted treatment duration and low concurrent chemotherapy utilization.

## Teaser

Patterns of brachytherapy utilization in Ethiopia with characteristics of treatment of 138 patients with cervical cancer.

## INTRODUCTION

Cervical cancer is the second most commonly diagnosed cancer in Ethiopia and the leading cause of cancer death in women, with an estimated 7,445 newly diagnosed cases and 5,338 deaths every year.^1^ Although cervical cancer is considered nearly completely preventable because of the highly effective primary (HPV vaccine) and secondary (screening) prevention measures, women in sub-Saharan Africa largely lack such preventive measures, with only 16.9% ever screened.^2^ Hence, most patients with cervical cancer in the region seek health care at an advanced stage when treatment options are limited.^3^ This is compounded with insufficient infrastructures and lack of expertise to perform oncologic resections as well as to deliver radiation treatment for these patients. Accordingly, the treatment of invasive cervical cancer continues to be a major challenge in many sub-Saharan African countries because of the lack of surgical facilities, skilled providers, and radiotherapy (RT) services.^4^

**CONTEXT**

**Key Objective**
Brachytherapy (BT) was not a component of treatment of patients with cervical cancer in Ethiopia until 2015. This paper describes the patterns of BT utilization during the first 3 years of implementation as well as presents characteristics of 138 patients with cervical cancer treated with curative intention of treatment.
**Knowledge Generated**
The utilization of BT service increased gradually over the 3-year period and enabled the delivery of >80 Gy radiotherapy (RT) to patients with cervical cancer (mostly stage IIB). However, there was a protracted overall treatment duration of >8 weeks and very low concurrent chemotherapy utilization in a number of these patients.
**Relevance**
The paper provides foundation for implementation of a quality assurance activity in the treatment of cervical cancer in Ethiopia as well as other programs in the region while implementing BT. Moreover, it demonstrates the urgent need for more RT facilities equipped with BT in Ethiopia.

Concurrent chemoradiation and brachytherapy (BT) represent the standard treatment for locally advanced cervical cancer. Therefore, BT is a vital component of the curative treatment of locally advanced disease.^5^ The National Comprehensive Cancer Network (NCCN) Harmonized Guidelines for sub-Saharan Africa, which were adopted by the Ethiopian Ministry of Health, recommend chemoradiation with external-beam RT (EBRT) followed by a boost RT dose delivered by BT for the treatment of locally advanced cervical cancer.^6^ Boost dose with BT enables to enhance the dose to the tumor while sparing the surrounding healthy tissue. The practice of BT has evolved over many decades from an orthogonal two-dimensional (2D) X-ray imaging–based technique to the use of image-guided BT using computed tomography (CT) or magnetic resonance imaging, which has enabled dose adaptation to the tumor as well as dose escalation.^7,8^

In an effort to improve the care for patients with cervical cancer in Ethiopia, the first high-dose-rate (HDR) cobalt-60 intracavitary BT machine with a 2D treatment planning technique has been in service since July 2015. Before the implementation of BT, boost RT to patients with cervical cancer in Ethiopia were delivered using a 2D EBRT. In this report, we have assessed the pattern of utilization as well as the practice paradigms in the management of cervical cancer patients with BT during the first 3 years of service. Hence, we will have a better understanding of the opportunities and challenges of implementation of this RT program in one of the largest populous countries in sub-Saharan Africa.

## MATERIALS AND METHODS

### Study Setup

The study was conducted at Tikur Anbassa Specialized Hospital (TASH) oncology unit, which was the only RT center in Ethiopia at the time of the study. During the study period, the center was equipped with one functional cobalt-60 teletherapy machine and one HDR BT machine dedicated for gynecologic malignancies. The center also has a daycare and inpatient units for chemotherapy administration.

### Study Patients

All women treated for invasive cervical cancer in the center since the commencement of BT service July 2015 to August 2018 were identified. Our study included only patients treated with curative intention and who were able to complete the total dose of RT from both EBRT and BT. All patients were staged using the 2018 International Federation of Gynecology and Obstetrics (FIGO) staging system with physical examination, ultrasound to rule out hydronephrosis and liver metastases, and chest X-rays to detect lung metastases.^9^ Staging CT scans were used when chest X-ray or ultrasound findings were ambiguous, but were not routine.

### Treatment Modalities

Concurrent chemo-RT with EBRT followed by BT boost with cisplatin 40 mg/m^2^ once per week for 5 weeks is the standard of treatment adopted by the department in the treatment of locally advanced cervical cancer.

#### EBRT Technique

EBRT was delivered using a cobalt-60 teletherapy machine. In this first phase of RT, a total dose of 40-50 Gy in 2-Gy fractions was given to the whole pelvis either by anteroposterior (AP/PA) fields or box technique on the basis of patient AP separation (box technique for a patient with >20 cm AP separation while using cobalt-60 teletherapy). Treatment planning was performed on the basis of bony landmarks without simulator radiographs. Treatment volumes consisted of whole pelvic RT extending superiorly from the L4-L5 intervertebral space to the ischial tuberosity inferiorly, but 3 cm below any palpable disease in the lower vagina, and 2 cm lateral to the pelvic brim. In the box technique, the superior, inferior, and later border stayed the same as AP/PA field, while the anterior border is placed anterior to the symphysis pubis bone and the posterior border is S2/S3 sacral bone for early stage and whole sacral bone for stage IIIB/IVA. Also, for a maximal sparing of the bowel and normal bone structures, custom blocks were placed on the AP field at the level of the iliac crest and the femoral heads. Once the first phase is complete, tumor response and feasibility of phase two boost RT with BT was made by clinical examination.

#### BT

Treatment was delivered using Eckert & Ziegler MultiSource remote afterloader HDR BT machine which uses Cobalt-60 radiation source. Each application of intracavitary BT was performed using an opioid analgesic (pethidine 50 mg-100 mg intramuscular) without sedative anesthesia. We used different size ring applicators with intrauterine tandem for intact uterus and vaginal cylinder applicator for post-hysterectomy patients. The tandem and ring applicators were from Eckert & Ziegler BEBIG (Eckert & Ziegler group, Belgium) and the available applicator angles were 30°, 45°, and 60°; available ring diameters were 25, 30, and 35 mm, and accessible tandem lengths were 20, 40, and 60 mm. The choice of the applicator angle, ring diameter, and tandem length for the first fraction were decided on the basis of patient anatomy. Similarly, variable cylinder lengths and diameters from Eckert & Ziegle BEBIG were used to adapt to the patient's anatomy. A Foley catheter was inserted into the bladder and inflated with 7-mL urografin diluted in normal saline and pulled down to localize the bladder point. Once the applicator is in place, gauze packing was applied to stabilize the applicator and to push away the rectum and bladder from the applicator in ring and tandem application.

#### Dosimetry

After the completion of applicator insertion, orthogonal radiographs (AP and lateral views) of the patients were taken using a C-arm X-ray machine. The images were then transferred onto HDRplus treatment planning software for dose calculations. A 2D treatment planning was made on the basis of a standard loading pattern. The dose prescription point, point A (a para-cervical reference point), and the organs at risk points (bladder and rectal points) were identified using the International Commission on Radiation Unit and Measurement (ICRU) 38 recommendations. In those patients treated with a cylinder applicator, the cylinder diameter used ranged from 2 to 4 cm and treatment length ranged from 3 to 8 cm, and the prescription point was at the cylinder surface. A systematic prescription of 4 × 6.5 Gy was normalized to points A or to the cylinder surface to deliver a total equivalent dose in 2 Gy fractions (EQD2) dose of >80 Gy from both EBRT and BT. The dose from each HDR-BT fraction is converted into EQD2 considering α/β = 10 Gy for the tumor and α/β = 3 Gy for the organ at risks. As a standard department practice, the cumulative RT dose from EBRT and BT to bladder and rectum should not exceed 80 and 75 Gy, respectively.

### Data Collection and Statistical Analysis

Patient data were gathered by reviewing paper charts and retrieving from BT planning computers. Descriptive statistics were computed using SPSS 22 (IBM, Armonk, NY).

### Ethics

Ethical approval for the study was obtained from the departmental review boards of Addis Ababa University School of Medicine Department of Clinical Oncology.

## RESULTS

### Patient Characteristics

A total of 207 patients were identified to receive BT during the first 3 years of service. However, 69 patients were excluded as they were either not given a curative intention total dose of RT or did not complete the planned treatment, and a total of 138 patients were included in the final analysis of this study. In this cohort of 138 patients with cervical cancer treated with BT with a curative potential of treatment during the first 3 years of BT service at the sole RT center in Ethiopia, both the mean and median age was 50 (range, 22-75 years). All the patients were staged according to the 2018 FIGO staging system, and were in stage Ib-IIIb group. Stage IIb was found to be the predominant, 66.4% (n = 85). Additionally, 21% (n = 29) had undergone hysterectomy (unspecified type) previously and upstaged during pathologic specimen evaluation. Overall, the commonest histologic type was squamous cell carcinoma (92.8%; n = 128). Patient characteristics are listed in Table 1.

**TABLE 1:**
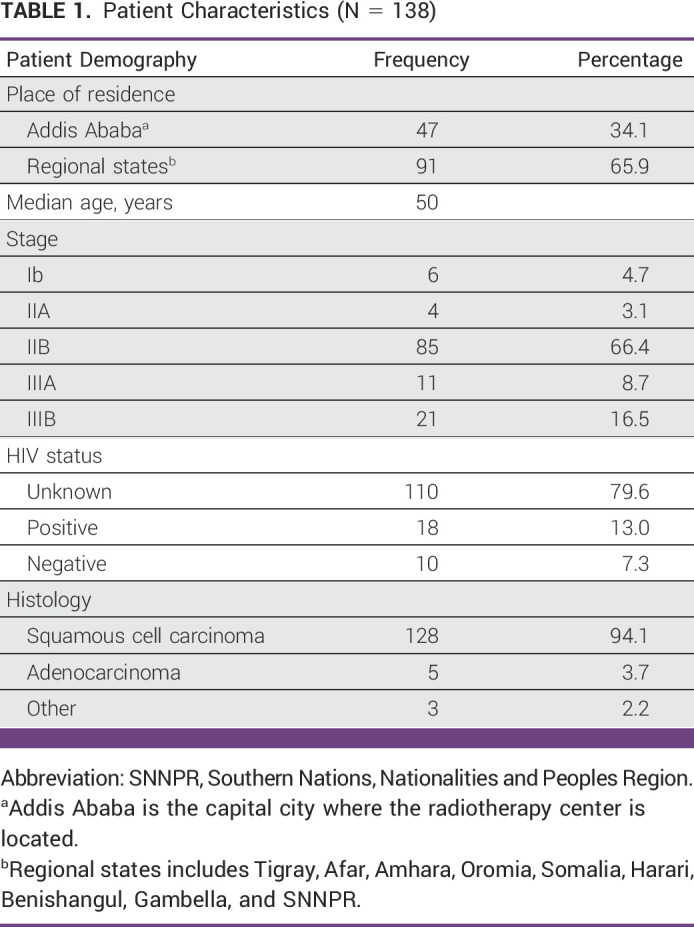
Patient Characteristics (N = 138)

### Patterns of BT Utilization

**FIG 1:**
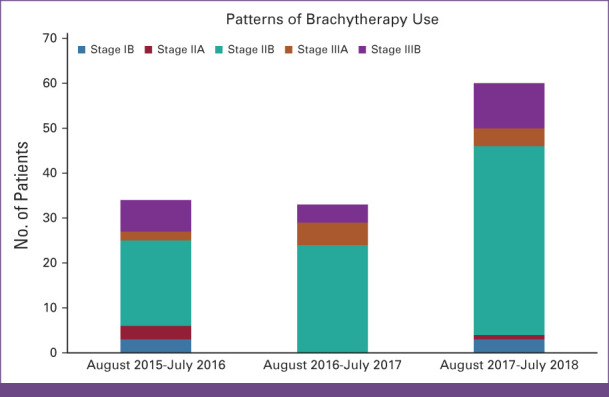
Patterns of brachytherapy utilization divided by year and stage.

During the first 3 years of commencing the BT service at the center, that is, between July 2015 and August 2018, in each year, n = 37, n = 36, and n = 65 patients received BT for a boost RT after EBRT with a curative potential of treatment. The patterns of BT utilization divided by year and stage are shown on Figure 1.

### Treatment Characteristics

The median waiting time for EBRT was 5 months in a range of 1-25 months. The median time gap between EBRT and BT was 8 days with a range of 1-35 days. The overall treatment time duration including both EBRT and BT was >8 weeks in 21% (n = 29) of the patients with a range of 6-11 weeks.

During EBRT, a RT dose of 40–50 Gy followed by a 5–8.5 Gy dose of BT three to six times were given to these patients, resulting in a total dose of RT in EQD2 in the range of 76-86 Gy. However, an EBRT dose of 46 Gy in 23 fractions followed by a 26-Gy BT in four insertions as per the department protocol was given for n = 86 (62.3%) patients. The median dose to point A was 6.5 Gy, while the median dose to organ-at-risk points bladder and rectum was 79 and 74 Gy, respectively. The dose received by the organ-at-risk bladder point was >80 Gy in 6.5% (n = 9) of the patients. The dose received by the organ-at-risk rectal point was >75 Gy in 3% (n = 4) of the patients.

Although the department's cervical cancer treatment protocol recommends concurrent chemo-RT with platinum agent in the treatment of a locally advanced cervical cancer, concurrent chemotherapy was given only for 35% (n = 50) of the patients for an undocumented reason. Data regarding dosing and number of chemotherapy cycles given were also incomplete in the patients receiving concurrent chemotherapy. Patient treatment characteristics data are summarized in Table 2.

**TABLE 2:**
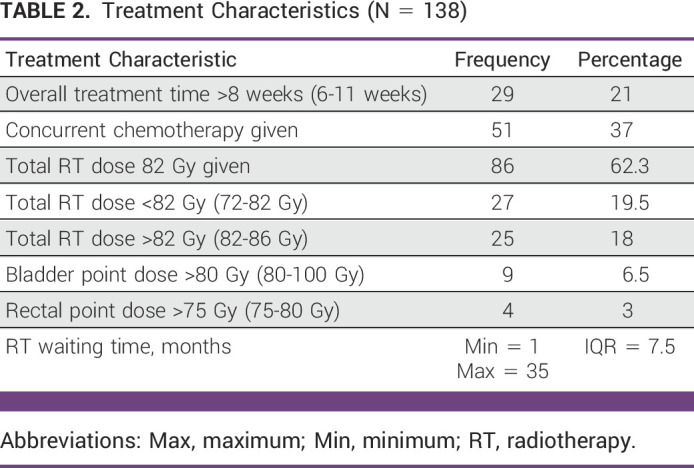
Treatment Characteristics (N = 138)

## DISCUSSION

This cross-sectional study on the characteristics and pattern of BT utilization in the treatment of cervical cancer in Ethiopia is, to our knowledge, one of the few descriptive analyses of BT use in sub-Saharan Africa. The most substantial finding of our study is an increasing utilization of BT in the treatment of Ethiopian cervical cancer patients with curative potential. This is significant because in previous studies from Ethiopia that reported on the outcome of patients who received oncologic therapy for cancer of the cervix uteri, one of the limitations stated was the absence of BT in the RT delivery.^3^ The advantage of BT especially for early and locally advanced cervical cancer was noted in different studies emphasizing the benefit of delivering high dose to cervix and lower dose to organ at risks such as rectum, bladder, and small intestine. Primary tumor remission rate, recurrence rate, and all types of survival rates were improved in the BT group.^10^ BT is important to achieve sufficient doses to the periphery and the central part of the tumor, and should always be considered in treatment of cervical carcinomas.

In our cohort of 138 patients, the median age at diagnosis was 50 years and FIGO stage IIB was the commonest stage at diagnosis, which is consistent with a previous report on cervical cancer in Ethiopia in which the median age at diagnosis was 49 years and FIGO stage IIB-IIIA at diagnosis was the commonest stage.^3^ However, as this cohort of patients are those to have received a curative intention of treatment, the stage distribution in this study is not representative of the overall distribution of cervical cancer in the country. Moreover, the waiting times until the start of RT from the date of diagnosis was very long (median, 5 months) and is thought to be a possible factor for stage migration and exclusion from treatment with a curative potential. As expected, the prevalence of HIV in this cohort was much higher than that of the general Ethiopian population (13% *v* 1.5%), which has also been reported as 9% in the study by Kantelhardt et al.^3^ Studies from other sub-Saharan African countries also reported such a higher-burden HIV prevalence in patients with cervical cancer.^11^ However, not all patients in our cohort were screened, thus the true rate may have been even higher. We encourage screening all patients with cervical cancer for HIV infection as well as lay out a reasonable ground for the ongoing effort in Ethiopia to screen HIV-positive women for cervical cancer.^12^

In our cohort of patients, the overall treatment time duration including both EBRT and BT was >8 weeks in 21% (n = 29) of the patients with a range of 6-11 weeks. Although treatment outcomes were not assessed in our study, it is recommended to finish the overall treatment in less than 8 weeks since other studies have shown each extra day to result in an approximately 0.5%-1% decrease in pelvic control and cause-specific survival.^13,14^ Given a variety of reasons for prolongation of treatment (patient and health system side), a considerable proportion of patients were managed on time. Still, measures are needed to assure guideline-concordant therapy for all patients such as monitoring timeliness over time. Majority of patients in our study group (62%, mostly stage IIb) received a total RT dose of 82 Gy from EBRT and BT boost. Before the implementation of BT, delivering such a high dose of RT to the pelvis using the available 2D EBRT alone was not feasible and therefore usually limited to <66 Gy.^15^ However, the NCCN Harmonized Guidelines for sub-Saharan Africa recommend an RT dose ≥85 Gy in the treatment of such a group of patients with locally advanced cervical cancer.^6^ Thus, treatment optimization in these patients is still mandatory. This may include managing side effects, optimizing logistics, and assessing and searching solutions for patient's perceived barriers (access, affordability, accommodation, and acceptability). Additionally, audits of patient survival and other measures of treatment outcome would help to measure the success of this treatment paradigm.

Moreover, although all the patients were within a locally advanced stage group (Ib-IIIb), less than half of the patients (35%) were given concurrent chemotherapy for undocumented reasons. The possible explanations for not delivering concurrent chemotherapy are lack of hospital beds and chemotherapy availability, which is a common challenge in sub-Saharan Africa.^16^ Additionally, comorbidities and side-effect management is challenging with limited resources. Furthermore, this is compounded by the inadequate documentation of the dose and number of cycles of the chemotherapy given as seen in this study. However, studies have shown that the use of concurrent chemo-RT results in a 30%-50% decrease in the risk of death compared with RT alone.^17^ Additionally, a meta-analysis has reported that chemo-RT leads to a 6% improvement in 5-year survival.^18^ The low utilization of concurrent chemotherapy seen in the treatment of these patients could be potentially addressed by implementation of regular quality control activities.

In our cohort of patients, rectal and bladder doses were estimated using the ICRU reference points.^19^ As a standard institutional practice, bladder and rectum point doses were kept to <80 and 75 Gy, respectively. However, the RT dose received by the organ-at-risk bladder point was >80 Gy in 6.5% (n = 9) of the patients and on the rectal point, it was >75 Gy in 3% (n = 4) of the patients. Since studies have shown late side effects after RT for cervical cancer, including potential injury to bladder and rectum, consideration should be given to dose reduction to organs at risk and when feasible image-guided BT should be applied to optimize treatment delivery.^20,21^

In conclusion, the utilization of BT service in TASH increased gradually and the commencement of BT service has enabled the delivery of a higher RT dose for patients with cervical cancer, mostly stage IIB. However, protracted treatment duration of one in five patients and concurrent chemotherapy utilization rates of one in three patients only are some of the service challenges noted in this study. Studies are needed to identify patient and health system factors that contributed to these guideline deviations. Although there is an encouraging pattern of utilization of BT, only a small proportion of patients with treatable cervical cancer in the country have access to this life-saving BT service. Thus, more RT facilities equipped with BT are urgently needed for patients presenting with curative disease.
